# An *In-silico* Screening Strategy to the Prediction of New Inhibitors of COVID-19 M^pro^ Protein

**DOI:** 10.22037/ijpr.2021.114997.15146

**Published:** 2021

**Authors:** Maryam Abbasi, Hojjat Sadeghi-aliabadi

**Affiliations:** a *Department of Medicinal Chemistry, Faculty of Pharmacy, Hormozgan University of Medical Sciences, Bandar Abbas, Iran. *; b *Department of Medicinal Chemistry, Faculty of Pharmacy, Isfahan University of Medical Sciences, Isfahan, Iran.*

**Keywords:** COVID-19, Mpro protein inhibitors, Molecular dynamic simulation, Induced fit docking, Binding free energy, Lasofoxifene

## Abstract

The coronavirus disease-2019 (COVID-19) was first recognized in Wuhan, China, and quickly spread worldwide. Between all proposed research guidelines, inhibition of the main protease (M^pro^) protein of the virus will be one of the main strategies for COVID-19 treatment. The present work was aimed to perform a computational study on FDA-approved drugs, similar to piperine scaffold, to find possible M^pro^ inhibitors. Firstly, virtual screening studies were performed on a library of FDA-approved drugs (43 medicinal compounds, similar to piperine scaffold). Among imported 43 drugs to virtual screening, 34 compounds were extracted. Four top-ranked drugs in terms of the highest interactions and the lowest binding energy were selected for the IFD study. Among these selections, lasofoxifene showed the lowest IFD score (-691.743 kcal mol^-1^). The stability of lasofoxifene in the COVID-19 M^pro^ protein active site was confirmed with 100 ns MD simulation. Lasofoxifene binding free energy was obtained -107.09 and -173.97 kcal mol^-1^, using Prime MM-GBSA and g_mmpbsa methods, respectively. The identified lasofoxifene by the presented computational approaches could be a suitable lead for inhibiting M^pro^ protein and COVID-19 treatment.

## Introduction

Coronavirus disease 2019 (COVID-19) is an emerging developed human infectious from coronavirus family, outbreak in Wuhan, China, and has been outspreading quickly in China and other countries since December 2019 (1). The coronavirus family comprises two high pathogenic forms; SARS-CoV (Severe Acute Respiratory Syndrome in 2002) and MERS-CoV (Middle East Respiratory Syndrome in 2012), but the COVID-19 has a significant difference from them due to the mutation processes (2). According to WHO reports, COVID-19 has infected more than 88 million and close to 2 million deaths worldwide (10 January 2021).

The main awful manifestations of COVID-19 are fever, cough, severe acute respiratory syndrome, and the lack of therapeutic protocols (3). 

The coronavirus family includes the single-strand, positive-sense RNA genomes having 6-12 open reading frames (ORFs). The first and largest ORF contains genetic codes for two polyproteins termed ppla and pplab, which are autoproteolytically cleaved into 15 or 16 nonstructural proteins (nsp) (4). Among these nsps, a chymotrypsin-fold proteinase, the main protease (M^pro^), plays a prominent role in viral gene expression and replication (5). 

The coronavirus M^pro^ protein is exceptionally conserved among the coronavirus family. M^pro^ dimer consists of 3 structural domains; domain I (residues 8-101), domain II (residues 102-184) have an antiparallel β-barrels, and domain III (residues 201-303) that is linked to domain II using a long loop region (residues 185–200). COVID-19 M^pro^ has a cysteine-histidine catalytic dyad, and its binding site is placed in a gap between domains I and II. Unlike other viral proteases, M^pro^ utilizes cysteine amino acid instead of serine for nucleophilic attacks in the active site (6); thus, cysteine-histidine catalytic dyad inhibition can stop virus function. 

Due to increasing concerns over the fast spread of COVID-19 globally, the rapid identification of drug candidates is essential. Hence, drug repurposing seems to be very impressive to create potent drugs to battle coronavirus in a short time (5). In addition, high throughput screening of all approved drugs on M^pro^ protein is a costly and time-consuming process; thus, high throughput virtual screening method could be an excellent alternative to save time and money. 

Recently, some dietary molecules from edible herbs and vegetables such as piperine, apigenin, curcumin, quercetin, and genistein, previously known as anti-viral, were evaluated as anti-COVID-19 agents (7). Their binding energy values in the COVID-19 M^pro^ protein active site were calculated using the molecular docking method. Among those, chromene scaffold demonstrated the lowest binding energy in EGCG (-6.99 kcal/mol), myricetin (-5.38 kcal/mol), and quercetin (-5.29 kcal/mol) compounds. Curcumin was another compound that showed low binding energy in COVID-19 M^pro^ active site (-6.04 kcal/mol). Piperine was another compound with a -5.16 kcal/mol binding energy in the M^pro^ protein active site. The virtual studies based on quercetin and curcumin scaffolds have been performed with COVID-19 M^pro^ protein (8, 9). 

**Figure 1:**
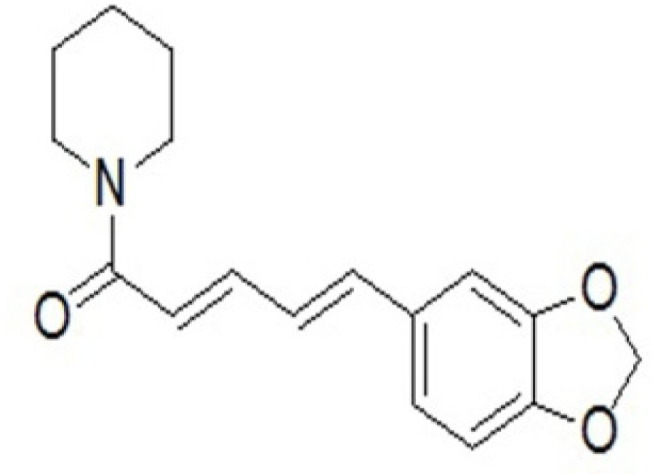
The chemical structure of piperine

Some FDA-approved drugs similar to the piperine scaffold (Figure 1) were extracted as a drug library in this study. Then virtual screening was performed on the drug library to find potential drugs as COVID-19 M^pro^ inhibitor. Induced fit docking and MM-GBSA calculation were performed to consider ligand and receptor flexibility and computing the binding free energy. Also, a molecular dynamic simulation was employed to confirm the stability of the best-chosen compound as anti-COVID-19 in the dynamic environment. 

## Experimental

*Creating data library*

The FDA-approved drugs (43 medicinal compounds) with at least 40% similarity with piperine scaffold were extracted from Drug Bank (www. drug bank. ac). The 3D structure of all compounds was drawn in Marvin Sketch v5.7, ChemAxon (10), and kept in a PDB format. 

*Docking simulation study*

Forty-three drugs were subjected to molecular docking studies. The molecular docking process was steered using the AutoDock4.2 software package (11). The PDB file of M^pro^ protein was obtained from the Protein Data Bank (PDB ID 6lu7) (6). Protein preparation was started with the elimination of all water molecules, ligands, and ions. After affixing polar hydrogens, the partial atomic charge was computed by the Kollman method, and the pdbqt file was kept (10). The pdbqt file of ligands was created by calculating the Gasteiger-Marsili charge (12). A grid box with the dimension of 50 × 50 × 50 Å (x, y, and z) was expanded on the protein binding pocket with a 0.375 nm spacing for each dimension; then, grid maps were made by Autogrid 4.2. 

All docking parameters were unchanged except for the number of Lamarckian job, the initial population, and the maximum number of energy evaluations, respectively 50, 150, and 25 × 10^5^. Finally, the docking process was performed by AutoDock 4.2. Docking results were analyzed in terms of conformation and binding energy in the active site of the M^pro^ protein. The best drugs with the lowest binding energy and the highest interaction in the binding pocket were chosen. The drug-protein interactions were imagined using AutoDockTools 1.5.6 and PyMOL Tcl (13). 

*Induced fit docking*

*Protein structure preparation*

The atomic coordinates of M^pro^ protein were obtained from the RCSB protein data bank (PDB ID 6lu7) and were prepared using Schrodinger’s protein preparation wizard module (14, 15). The M^pro^ protein was provided by removing crystallographic water molecules, adding all hydrogen atoms to protein, allocating bond orders, generating disulfide. To process the maximum degree of hydrogen bond interactions, 180° spins of the terminal angle of Asn and Gln amino acids were allocated. Finally, a restrained minimization was carried out using optimized potentials for liquid simulations (OPLS-2005) force field to optimize the geometry and minimize the energy of the M^pro^ protein (16). After energy conversion, the minimization was done, and the RMSD score got a maximum cut-off of 0.420 Å. 

*Compounds preparation*

The 3D structure of compounds was optimized using the LigPrep part of the Schrodinger suite 2015 (17). The most probable ionization state was generated at the cellular pH value of 7.4 using the Epik tool (18). Finally, the compound with the lowest energy conformer was optimized using the OPLS-2005 force field (16). 

*Induced fit docking*

Induced fit docking (IFD) is a powerful and precise method to compute ligand and receptor flexibility (19). To understand the protein-ligand interactions of the docked compound, we performed the induced fit docking in the Schrodinger software suite (20). The IFD protocol was carried out in three successive steps. Firstly, optimized ligand and rigid protein were introduced to the IFD module. The docking box was specified to include all residues within the radius 30 Å from the ligand center. A van der Waals scaling of 0.7 and 0.5 was utilized for non-polar atoms of the protein and ligand, respectively. The Glide SP mode did the initial docking, and 20 ligand postures were maintained for protein structural modifications. Secondly, each of the ligand poses was subjected to side-chain and backbone refinement. All residues with at least one atom within 5 Å of each corresponding ligand pose were inserted into a conformational search and minimization using the Prime program (15). The refined complexes were arranged by prime energy. Finally, the new receptor-ligand conformations within 30 kcal/mol of minimum energy were introduced to Glide redocking. The more desirable the binding affinity identified with, the more negative the Glide Score. The visualization of the best pose of protein-ligand complexes was done using PyMOL Tcl.

*MM-GBSA calculation*

The best obtained ligand-M^pro^ complex from IFD was further investigated with MM-GBSA procedure in the prime segment of Schrödinger suite 2015 (15). MM-GBSA technique is introduced as a valuable and effective method to computing the binding free energy (ΔG_bind_) between ligand and receptor with more precision. In this method, molecular mechanical (MM) energies are considered with a continuum solvent generalized Born (GB) model for polar solvation and a solvent-accessible surface area (SASA) for non-polar solvation (21). The binding free energy of the docked ligands is calculated according to the following equations (22, 23):

${∆G}_{\mathrm{binding}}={∆G}_{\left(\mathrm{complex}\right)}-{(∆G}_{\left(\mathrm{protein}\right)}+{∆G}_{\left(\mathrm{ligand}\right)})$ Equation 1.

$∆G={∆E}_{\mathrm{MM}}+{∆G}_{\mathrm{solvation}}$ Equation 2.

${∆G}_{\mathrm{solvation}}={∆G}_{\mathrm{GB}(\mathrm{solvation}-\mathrm{electrostatic})}-{∆G}_{\mathrm{SA}(\mathrm{nonpolar})}$ Equation 3.

$E_{\mathrm{MM}}=E_{\mathrm{internal}}+E_{\mathrm{electrostatic}}+E_{\mathrm{vdW}}$ Equation 4.

$E_{\mathrm{internal}}=E_{\mathrm{bond}}+E_{\mathrm{angle}}-E_{\mathrm{torsion}}$ Equation 5.

Where ${∆E}_{\mathrm{MM}}$is the difference of the gas phase of MM energy between the M^pro^-ligand complex and the aggregate of the energies of the free ligand and M^pro^ protein. In the end, the outcomes were ordered based on the calculated ${∆G}_{\mathrm{binding}}$Values. 

*Molecular dynamics simulation*

The finest ordered candidate from docking outcomes was further investigated for appraising their thermodynamic properties and the binding stability in the M^pro^ binding pocket by molecular dynamic (MD) simulation studies. The molecular dynamic simulation was carried out by the GROMACS-2019.3 package (24, 25). The Amber99.sb force field was employed in MD simulations (26), and all drug topology parameters were ready by the AnteChamber Python Parser InterfacE (ACPYPE) (27). The pKa values were calculated to determine which residue adopts non-standard ionization states, using PROPKA 3.1 webserver (28). A TIP3P water model was chosen (29), and the drug-protein complex was waterlogged in a dodecahedron box. To neutralize the system, Na^+^ ions were substituted with solvent water molecules. The complex energy minimization was carried out, and two stages of the process started MD simulation: 1) 500 ps simulation in the NVT ensemble (constant number of particles, volume, and temperature); 2) 1 ns simulation in the NPT ensemble (constant number of particles, pressure, and temperature). In the end, the MD simulation was run at 300 K temperature for 100 ns. The Particle Mesh Ewald (PME) method and the linear constraint (LINCS) algorithm were employed to calculate long-range electrostatic interactions and covalent bond constraints, respectively. Moreover, structure imagining was done by VMD 1.8.6 (30) and PyMOL Tcl. 

## Results and Discussion

*Virtual screening study on FDA approved drugs*

**Figure 2:**
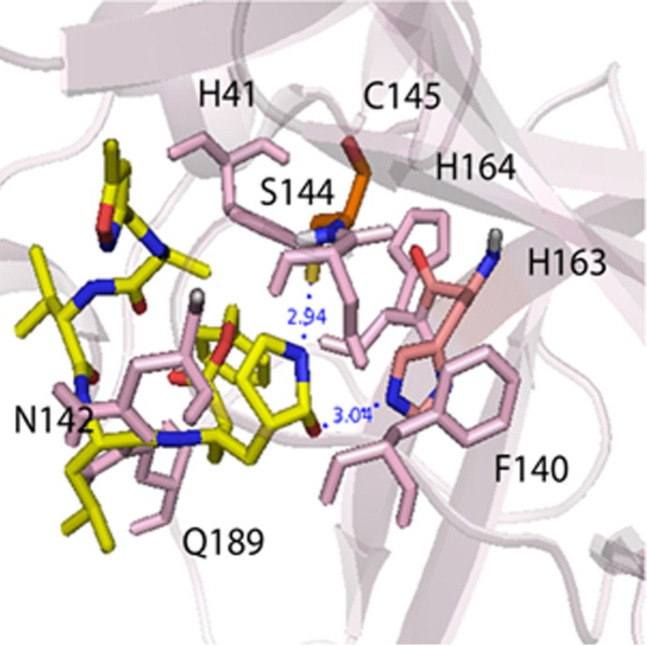
The main interactions between **N3** ligand and M^pro^ protein

The validation docking was carried out and interactions between **N3 **(N-[(5-methylisoxazol-3-yl)carbonyl]alanyl-L-valyl-N-1-((1R,2Z)-4-benzyloxy)-4-oxo-1-{[(3R)-2-oxopyrrolidin-3-yl]methyl}but-2-enyl)-L-leucinamide, crystallography ligand) and M^pro^ protein was compared with the crystallographic poses in PDB: 6lu7. The binding pocket of **N3** was investigated as the following: the catalytic His163 and Cys145 residues in the bottom of the packet formed two hydrogen bonds, and hydrogen bonds were seen between Gly143 and Glu166 amino acids in the edges pocket with **N3. **The main hydrophobic amino acids that sat around **N3** were His41, Phe140, Lue141, Asn142, Ser144, His164, Met165, His172, and Gln189, as seen in Figure 2 (6). The root-mean-square deviation (RMSD), between the valid conformations and its crystallography conformation, was obtained below 2 Å. 

To predict new M^pro^ inhibitors, a structure-based virtual screening (VS) study was carried out over created library from the Drug Bank web server. Docking runs were done for all 43 drugs similar to the piperine scaffold, and its results were organized in terms of binding energy and fundamental catalytic interactions. Thirty-four of them were sat in the M^pro^ active site, but between them, four drugs (curcumin, lasofoxifene, alvimopan, and donepezil) with the lowest binding energy were selected for further investigation. Among these four drugs, curcumin structurally showed the most similarity to the piperine scaffold. In lasofoxifene, alvimopan, and donepezil structures, saturated rings are piperidine or pyrrolidine rings, the same as piperidine ring in piperine structure.

Curcumin, extracted from a plant called “turmeric,” with the lowest binding energy, acts as a lipoxygenase inhibitor and prevents tumor invasion by irreversibly binding CD13/aminopeptidase. Turmeric is commonly consumed as a color food, and its root is also used in a few medicinal products to treat pain and inflammation, such as osteoarthritis. The medicinal properties of turmeric have been known for thousands of years, but curcumin from natural dietary or synthetic sources gained first approval by the Food and Drug Administration (FDA) in 2013. 

Lasofoxifene is a selective estrogen receptor modulator (SERM) with a non-steroidal structure that selectively binds to estrogen receptors α and β. The European Commission approved it in March 2009. Lasofoxifene could be used for postmenopausal osteoporosis treatment to decrease the risk of both vertebral and nonvertebral fractures. 

Alvimopan competitively binds to the μ-opioid receptor as a selective antagonist. FDA licensed alvimopan in 2008 to speed up the time to upper and lower gastrointestinal recovery following surgeries. 

Finally, donepezil increases the accessibility of acetylcholine at the synapses by binding reversibly to acetylcholinesterase and inhibiting the hydrolysis of acetylcholine. Donepezil gained approval by FDA in 2004 and could be used to treat confusion (dementia) related to Alzheimer’s disease. 

*Induced fit docking analysis*

Although molecular docking simulation using Autodock software is highly favored in presenting the ligand poses within the protein active site, the protein is considered rigid in docking calculations. In this study, the IFD method was carried out to account for both ligand and receptor flexibility. 

**Figure 3:**
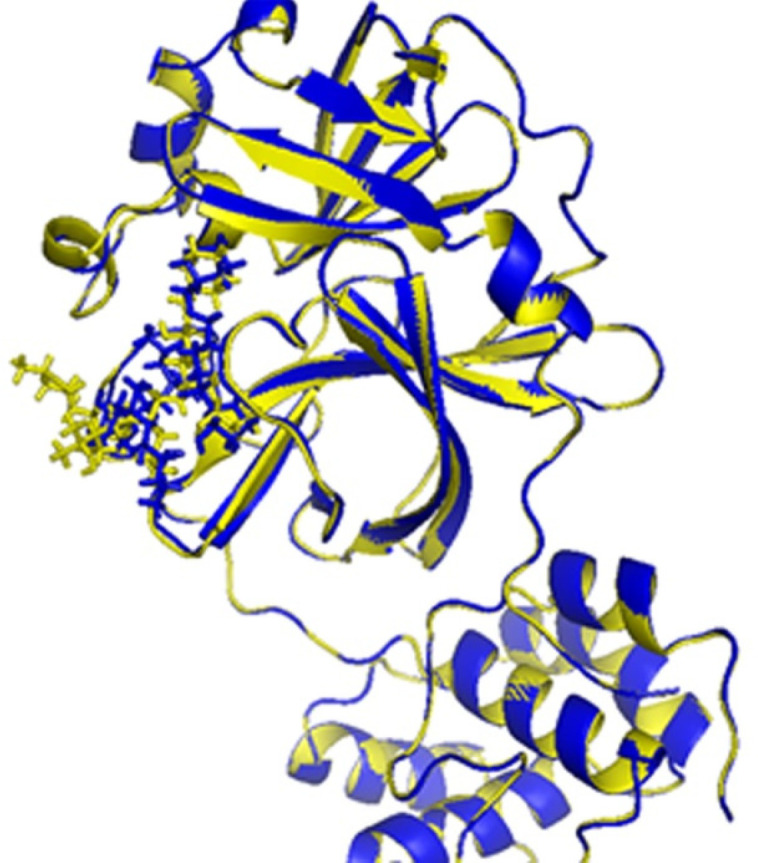
The binding sites of 6lu7 with ligand N3: native N3 (yellow), IFD-generated N3 model (blue).

The validation of the IFD model was carried out before the docking simulation. The IFD-generated **N3** model (cyan, Figure 3) and the native structure of **N3** (yellow, Figure 3) in N3/M^pro^ complex (6lu7) were superimposed, and RMSD of 2.34 Å was obtained for entire heavy atoms (excepting the hydrogen atoms). Therefore, the IFD module could be predicting the binding interactions between inhibitors and M^pro^ protein. 

**Figure 4:**
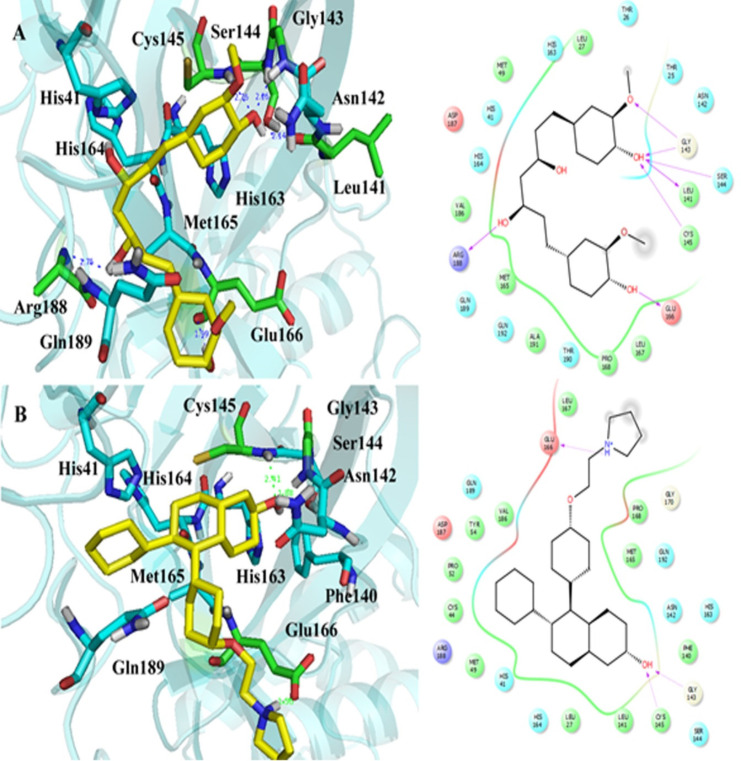
The IFD generated interactions. A) 3D and 2D interactions between curcumin and covid-19 M^pro^ protein; B) 3D and 2D interactions between lasofoxifene and covid-19 M^pro^ protein

IFD was performed between M^pro^ protein and the four obtained drugs with the lowest binding energy by Autodock. The IFD results are reported in Table 1. The GlideScores in the curcumin-M^pro^ and lasofoxifene-M^pro^ complexes were obtained lower than alvimopan and donepezil complexes. It suggests that the binding affinity of curcumin and lasofoxifene complexes is also lower than alvimopan and donepezil complexes, the same as the calculated binding affinity by Autodock. The IFD generated interactions between curcumin and lasofoxifene with M^pro^ protein are shown in Figure 4. IFD results show that curcumin interacted with Cys145, Gly143, Glu166, Ser144, Leu141, and Arg188 of M^pro^ protein via hydrogen bonds lasofoxifene formed hydrogen bonds only with Cys145, Gly143, and Glu166 amino acids. As seen in Figure 4B, Glu166, with a negative charge, sat close to pyrrolidinium ion (the distance: 1.58 Å), and an ion bond could be formed between negative and positive charges. The ion bond formation could be a reason for the lasofoxifene GlidScore value that is close to the obtained value of curcumin. The docking affinity of lasofoxifene (IFD score = -691.743) was better than curcumin, alvimopan, and donepezil with IFD scores of -684.229, -672.998, and -674.228, respectively. 

*Prioritization of IFD-studied compounds based on MM-GBSA method*

Combining more energy terms such as surface accessibility area and solvation energy with a suitable force field can make more satisfactory accuracy for the ligand binding energy computing (21). Thus for each four selected compounds, the Prime MM-GBSA method was done on the state with the bottommost obtained GlideScore from IFD studies. The calculated ΔG_bind_ of the compounds and the contribution of main energy components (coulomb, covalent, hydrogen bonding, lipophilic binding, the generalized born electrostatic solvation, and van der Waals) were reported in Table 2. The same as IFD results, lasofoxifene showed the lowest ΔG_bind_. This suggests that lasofoxifene is the most stable ligand in the M^pro^ protein binding pocket. In Table 2, the free energy components revealed that the lipophilic and van der Waals interaction energies (ΔG_Lipo_ and ΔG_vdW_) have the most significant contribution in the ligands binding energy. The binding energy contribution of the main amino acids in the active site is shown in Table 3. In M^pro^ protein active site, the contribution of lipophilic interactions is more than hydrogen bond interactions. The contribution of His163 and Glu166 was more than other amino acid residues in the binding pocket. 

*Molecular dynamic simulation analysis*

According to Table 1, lasofoxifene showed the lowest binding energy among all 34 extracted drugs with two main hydrophilic interactions (hydrogen bonds with Cys145 and His163). MD simulation of lasofoxifene was performed to ensure its stability in the binding pocket of M^pro^ protein. Recently curcumin was introduced as M^pro^ inhibitor (31); therefore, it was chosen as a reference and compared its interaction modes with lasofoxifene.

**Figure 5:**
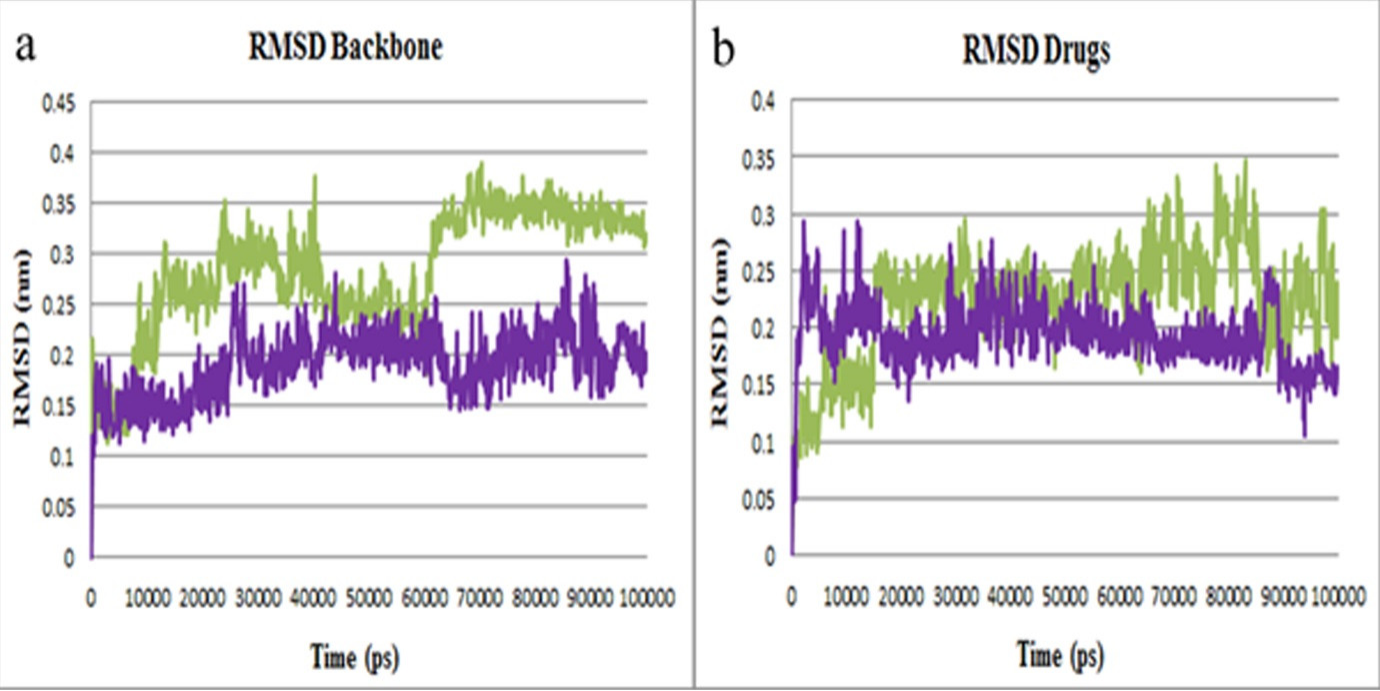
RMSD plots of M^pro^ protein inhibitors. (a) Backbone atoms RMSD of M^pro^-curcumin (purple) and M^pro^-lasofoxifene (green) complexes. (b) RMSD plot of curcumin and lasofoxifene

After 100 ns simulations, the MD trajectories were analyzed. RMSD was calculated to determine the conformational stability of M^pro^ protein in all simulation times. As illustrated in Figure 5a, the RMSD profile of backbone atoms in M^pro^-lasofoxifene and M^pro^-curcumin complexes showed small variations about 0.35 nm and 0.25 nm, respectively. By analyzing the RMSD plots of lasofoxifene and curcumin (Figure 5b), it can be recognized that both drugs were almost entirely superimposed during the simulation. RMSD plots suggest that both complexes were stable during simulation time. 

**Figure 6:**
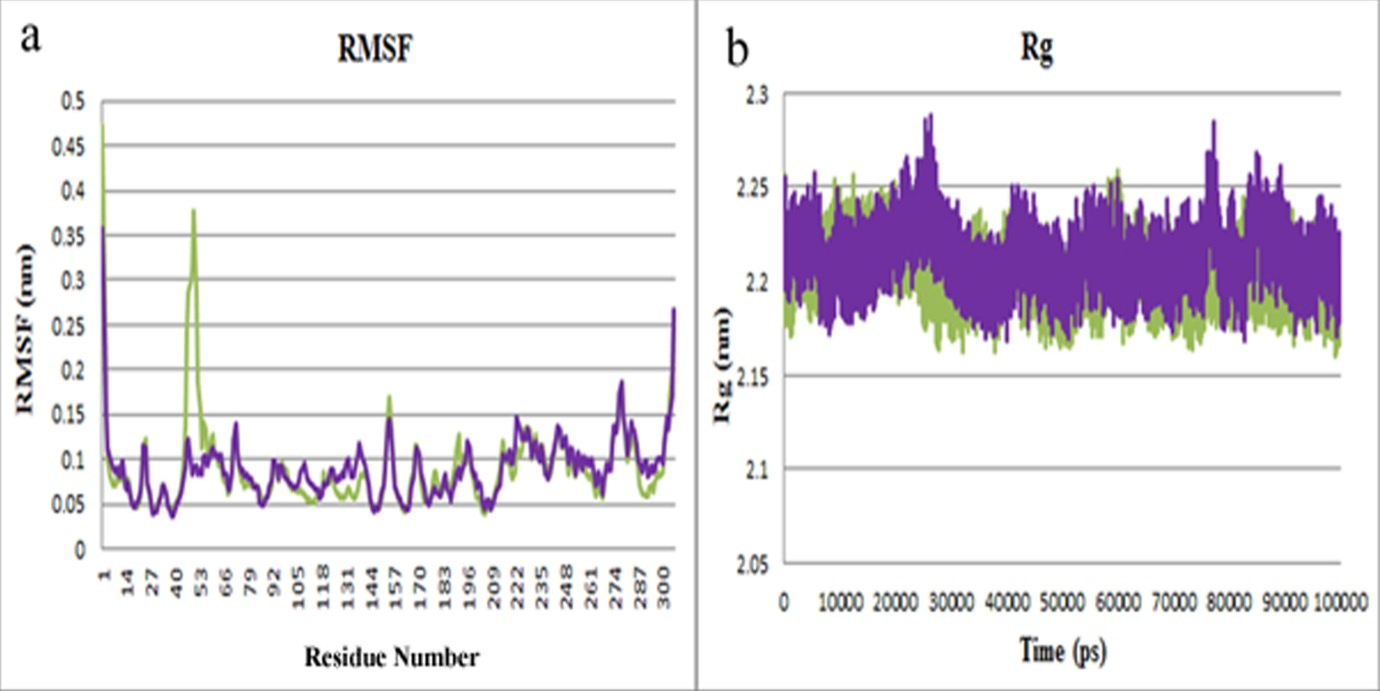
(a) The RMSF plot of M^pro^-curcumin (purple) and M^pro^-lasofoxifene (green).

Rg was calculated to evaluate the compactness of the protein. Variations of protein flexibility were obtained by RMSF, as are demonstrated in Figure 6. The oscillations of both complexes were superimposed (Figure 6a), except in residues 45-60 in the M^pro^-lasofoxifene complex. This indicates that the central regions of the protein, such as Cys145, His163, and His41, in both complexes were more stable during MD simulation. The Rg values of both complexes were identical, and their conjunction was kept in all simulation time, as represented in Figure 6b. Average values of RMSD, RMSF, and Rg were calculated at 0.225, 0.090, and 2.204, respectively, for M^pro^- lasofoxifene complex. The average values of RMSD, RMSF, and Rg of M^pro^- curcumin complex were also obtained from 0.193, 0.089, and 2.213, respectively. 

The binding free energy has also been recalculated for M^pro^-curcumin and M^pro^-lasofoxifene complexes using the g_mmpbsa program. The obtained average binding energy components are reported in Table 3. The results showed that M^pro^-lasofoxifene binding energy was lower than M^pro^-curcumin binding energy checked by both programs (MMGBSA and g_mmpbsa). The contribution of each main amino acid in the binding energy has shown in Table 4. 

**Table 1:** IFD results for the four obtained drugs with the lowest binding energy by Autodock

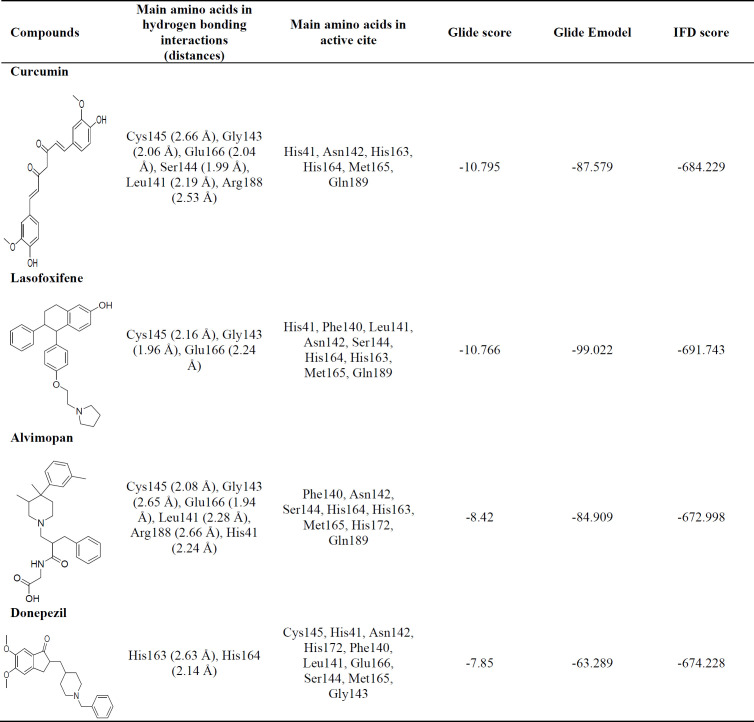

**Table 2:** The outcomes of binding free energy for the selected compounds using Prime-MM-GBSA calculations

Entry	∆G_Binding_	∆G_Coulomb_	∆G_Covalent_	∆G_Hbond_	∆G_Lipo_	∆G_SolvGB_	∆G_vdW_
**Curcumin**	-65.978	40.909	-25.380	0.012	-45.334	-3.162	-40.750
**Lasofoxifene**	-107.086	9.012	2.627	-0.298	-83.099	8.103	-49.746
**Alvimopan**	-50.841	-16.943	27.281	0.434	-50.572	18.863	-36.112
**Donepezil**	-49.525	23.517	4.184	-1.298	-40.009	-7.419	-36.127

**Table 3:** The main amino acids contribution to the binding free energy of the compounds, using Prime-MM-GBSA calculations

**Amino Acids**	**Compounds**	**Lipo Energy**	**H-bond Energy**	**Total Energy**
His41	**Curcumin**	-7.54	-0.31	-46.78
	**Lasofoxifene**	-6.27	-0.25	-45.78
	**Alvimopan**	-5.96	-0.11	-45.06
	**Donepezil**	-6.68	-0.15	-45.63
Leu141	**Curcumin**	-2.87	-0.36	-26.70
	**Lasofoxifene**	-3.05	-0.35	-26.05
	**Alvimopan**	-4.03	-0.36	-31.06
	**Donepezil**	-3.18	-0.35	-26.97
Gly143	**Curcumin**	-1.21	-0.21	-36.60
	**Lasofoxifene**	-1.26	-0.16	-37.78
	**Alvimopan**	-1.07	-0.01	-30.43
	**Donepezil**	-1.32	-0.18	-37.17
Ser144	**Curcumin**	-4.53	-0.60	-42.53
	**Lasofoxifene**	-4.41	-0.63	-41.38
	**Alvimopan**	-4.42	-0.67	-43.53
	**Donepezil**	-4.59	-0.60	-43.09
Cys145	**Curcumin**	-4.50	-0.24	-39.71
	**Lasofoxifene**	-4.51	-0.25	-40.00
	**Alvimopan**	-4.09	-0.13	-39.58
	**Donepezil**	-4.64	-0.14	-37.10
His163	**Curcumin**	-7.07	-0.38	-53.79
	**Lasofoxifene**	-6.96	-0.38	-54.58
	**Alvimopan**	-7.07	-0.40	-53.93
	**Donepezil**	-7.21	-0.38	-56.03
His164	**Curcumin**	-7.60	-0.25	-42.83
	**Lasofoxifene**	-7.52	-0.63	-40.14
	**Alvimopan**	-7.04	-0.27	-44.16
	**Donepezil**	-7.06	-0.27	-42.33
Glu166	**Curcumin**	-3.55	0.00	-54.26
	**Lasofoxifene**	-4.17	-0.01	-57.26
	**Alvimopan**	-4.80	-0.50	-59.28
	**Donepezil**	-3.53	0.00	-55.46

**Table 4:** The average binding energy components were obtained from the g_mmpbsa program

Complex	∆G_binding_^a^	∆G_polar_^b^	∆G_nonpolar_^c^	∆E_elec_^d^	∆E_vdW_^e^
**M** ^pro^ **-curcumin**	-81.392 ± 8.01	136.200 ± 6.24	-18.180 ± 0.34	-33.170 ± 3.99	-166.243 ± 8.99
**M** ^pro^ **-lasofoxifene**	-173.973 ± 11.97	145.290 ± 10.65	-15.324 ± 0.34	-157.672 ± 7.77	-146.267 ± 9.1

^a^Binding energy. ^b^Polar solvation energy. ^c^Non-polar solvation energy. ^d^Electrostatic component to the binding energy in kcal/mol.

^e^Van der Waals component to the binding energy in kcal/mol.

## Conclusion

Since inhibition of M^pro^ protein has been known as a critical strategy for COVID-19 treatment, highly effective M^pro^ inhibitors have been investigated with various in silico and traditional methods. In this study, virtual screening with different computational methods has been introduced to predict new M^pro^ inhibitors. According to this method, virtual screening was first performed on 43 drugs (similar to piperine scaffold). Finally, 34 medicinal compounds were extracted with therapeutic effects against osteoporosis, Alzheimer’s, cancer, and reduced heart rate. Among extracted drugs, curcumin, lasofoxifene, alvimopan, and donepezil demonstrated the lowest binding energy and the most interactions with the COVID-19 M^pro^ protein active site. Among extracted drugs, lasofoxifene, as a third-generation SERM with high selective affinity for both ERa and ERb subtypes, was the best in terms of binding energy and the critical interactions with amino acids. An induced-fit docking method was performed to consider the flexibility of both ligand and receptor. The obtained score from IFD confirmed molecular docking results, and the IFD score of lasofoxifene was observed lower than others (-691.743 kcal mol-1). Lasofoxifene binding free energy, calculated by two programs including Prime MM-GBSA and g_mmpbsa, showed the lowest value; -107.09 and -173.97 kcal mol-1, respectively. And finally, MD simulation investigations on the finest docked pose of lasofoxifene-M^pro^ and curcumin-M^pro^ complexes were employed to reveal the overall stability of the two complexes. RMSD, RMSF, and Rg results obtained from MD simulation revealed that lasofoxifene and curcumin exhibited the best stability in the M^pro^ active site. As a result, lasofoxifene or its close derivatives can be considered promising drugs for the treatment of COVID-19.

## Funding

We would like to express our special thanks to the Hormozgan University of Medical Sciences for their financial support (Project No. 990193).

## Declaration of competing interest

The authors declare no conflict of interest.

## Author contributions

Maryam Abbasi: Participated in the study’s design, carried out IFD study, performed molecular dynamic simulation, and drafted the manuscript. Hojjat Sadeghi-aliabadi: Participated in the study’s design, was responsible for the study registration and gave final approval of the version to be published. 
